# Liposomal β-Sitosterol Suppresses Metastasis of CT26/luc Colon Carcinoma via Inhibition of MMP-9 and Evoke of Immune System

**DOI:** 10.3390/pharmaceutics14061214

**Published:** 2022-06-07

**Authors:** Chao-Yu Shen, Chia-Fen Lee, Wei-Taur Chou, Jeng-Jong Hwang, Yeu-Sheng Tyan, Hui-Yen Chuang

**Affiliations:** 1 Department of Medical Imaging, Chung Shan Medical University Hospital, School of Medicine, Chung Shan Medical University, Taichung City 40201, Taiwan; shenchaoyu@gmail.com (C.-Y.S.); johnjjhwang@gmail.com (J.-J.H.); 2 Department of Radiology, Changhua Christian Hospital, Changhua 50006, Taiwan; mini124719@gmail.com; 3 Department of Medical Imaging and Radiological Sciences, Chung Shan Medical University, Taichung 40201, Taiwan; johnjjhwang@yahoo.com.tw; 4 Department of Biomedical Imaging and Radiological Sciences, National Yang Ming Chiao Tung University, Taipei 112304, Taiwan

**Keywords:** CT26/*luc* colorectal carcinoma, liposomal β-sitosterol, MMP-9, interleukin-12, interleukin-18, interferon-γ, bioluminescent imaging

## Abstract

β-sitosterol (SITO) has been reported with anticancer effects; however, with poor bioavailability. The current study aimed to investigate whether liposomal encapsulated β-sitosterol (LS) has a better inhibition effect on tumor metastasis than β-sitosterol in a CT26/*luc* lung metastasis mouse model and the possible underlying mechanism. LS was liposomal-encapsulated SITO and was delivered to mice by oral gavage. The cell viability was determined by the MTT assay, and invasiveness of the tumor cells and related protein expression were evaluated with the invasion assay and Western blotting. For therapeutic efficacy evaluation, male BALB/c mice were treated with PBS, SITO, and LS once a day for 7 days prior to intravenous injections of CT26/*luc* cells; treatments were continued twice a week post-cell inoculation throughout the entire experiment. Tumor growth inhibition was monitored by bioluminescent imaging (BLI). IL-12, IL-18, and IFN-γ in the intestinal epithelium were determined by ELISA. The results show that LS treatment had a better invasion inhibition with lower cytotoxicity than SITO when the same dose was utilized. Notably, mice treated with LS significantly exhibited fewer metastases to the lungs and other tissues/organs compared with the Control and SITO groups. Additionally, the IL-12, IL-18, and IFN-γ levels were significantly increased in the LS-treated mice compared with the Control and SITO groups. The underlying mechanism may be through the inhibition of MMP-9 and elicitation of the antitumoral Th1 immune response, such as increasing CD4^+^ and CD8^+^ T cells, IL-12, IL-18, and IFN-γ.

## 1. Introduction

Colorectal carcinoma is ranked the second among the most common causes of cancer death in 2020 worldwide, according to the WHO report [1]. Although early colorectal cancer has a >90% curative rate, the prognosis worsens rapidly once the cancer cells invade other organs, in which liver metastasis is the most often seen [2]. Invasion and metastasis are multistep processes involving altered adhesion molecule expressions and extracellular matrix (ECM) degradation [3]. The dysregulation of cadherin and zonula occludens-1 (ZO-1) strongly promotes the invasion and metastasis of colorectal cancer [4]. Adhesion molecules also regulate the trafficking, activation, proliferation, and target recognition of immune cells, affecting tumor progression [5]. β-sitosterol (SITO), also known as sitosterol, a type of phytosterol, has been shown to exert anti-inflammatory [6] and cholesterol-lowering [7] effects in preclinic studies. Additionally, SITO has been shown to trigger apoptotic cascades in various cancer cells [8,9,10].

SITO has been reported to regulate the immune responses in various diseases. 2,4,6-Trinitrobenzene sulfonic acid (TNBS)-induced colitis in mice could be suppressed by SITO through downregulating the expression of NF-κB-modulated proinflammatory cytokines, including TNF-α, IL-1β, and IL-6 [11]. It could also alleviate the sepsis process by inhibiting the NF-κB signaling pathway in a cecal ligation and puncture (CLP)-induced septic rat model [12]. SITO has also been shown to mitigate the proinflammatory responses caused by influenza A by inhibiting both RIG-1 and STAT1 pathways other than the NF-κB signaling pathway [13]. In addition, a combination of SITO and sitosterol glucoside was found to increase the IL-2 and IFN-γ levels, T-cell proliferation, and NK-cell activity in vitro and in vivo [14]. The antioxidant effects of SITO were found to protect lymphocytes from apoptosis in doxorubicin-treated mice [15] and repress the progression of melanoma in mice [16].

In order to increase the therapeutic gain, sometimes very high doses (up to 25–50 g per day) of free phytosterols are required due to its poor water solubility and bioavailability [17]. Liposomes are hollow spheres consisting of phospholipid bilayers enclosing an aqueous core and are widely applied as drug delivery vehicles. Liposomal drug delivery vehicles increase the drug stability, prolong the circulation time, and enable organ-specific uptake not only in living animals [18] but also in the clinic, such as liposomal doxorubicin for the treatments of several types of cancers. Several groups have synthesized liposomal drugs by replacing cholesterol with SITO and showed comparable drug incorporation, release and stability to cholesterol-based liposomes [19,20]. Here, we proposed to use liposomes as carriers to improve the bioavailability and anticancer effects on tumor progression and metastasis using colorectal carcinoma CT26/*luc* cells and the tumor-bearing mouse model.

## 2. Materials and Methods

### 2.1. Tumor Cell Line

Murine colorectal carcinoma CT26/*luc* cells stably expressing luciferase were utilized [21], and the correlation of emitted photons and cell numbers is shown in Supplementary Figure S1. CT26/*luc* cells were cultured in RPMI 1640 medium (Invitrogen, Carlsbad, CA, USA) supplemented with 10% FBS (Biological Industries, Kibbutz Beit HaEmek, Israel), 1% penicillin–streptomycin (Invitrogen), and 100 μg/mL G418 (Merck, Branchburg, NJ, USA). All cultures were maintained at 37 °C in a humidified incubator containing 5% CO_2_.

### 2.2. Preparation of Liposome-Encapsulated β-Sitosterol (LS)

Liposomal β-sitosterol (LS) was prepared by the thin-film hydration method with egg phosphatidylcholine (EPC, PC-98SR, NOF Corporation, Tokyo, Japan) and β-sitosterol (SITO, 83-46-5, Tama Biochemical Co., Ltd., Tokyo, Japan) in the mole ratio of 2:1. Both EPC and SITO were dissolved in chloroform in a round-bottom flask, and chloroform was evaporated at 40 °C with a rotovac machine to obtain a thin lipid film. The further evaporation was conducted to remove the residual chloroform. The lipid film was hydrated with phosphate-buffered saline (PBS) to adjust the concentrations of EPC to 50 µmol/mL.

The resulting liposomal suspension was heated to 50 °C and extruded through a 200-nm polycarbonate membrane filter (cat. no. GTBP01300, Merck KGaA, Darmstadt, Germany) using a Lipex Biomembranes Extruder (Vancouver, BC, Canada). Ten extrusion cycles were performed. Particle sizing was conducted by dynamic light scattering with Zetasizer Nano (Malvern Instruments, Malvern, UK). The phospholipid concentration of the liposomes was determined by a phosphorus assay based on the Rouser method [22].

### 2.3. Analysis of β-Sitosterol Concentration Using High-Performance Liquid Chromatography

Liposomal β-sitosterol (100 μL) and methanol (367 μL) were added into a 1.5-mL microcentrifuge tube, sonicated for 5 min, and centrifuged at 18,000× *g* for 5 min at 4 °C (Model 3700, Kubota, Japan). The pellet was dissolved in 550 μL methanol and 150 μL distilled water to make the SITO solution, and the mixture was centrifuged at 18,000× *g* for 5 min at 4 °C. The process was repeated twice to acquire the pure SITO, which was re-dissolved with 1 mL of methanol.

Analytical separation was performed by high-performance liquid chromatography (HPLC) with a Waters HPLC system (Waters Corporation, Milford, MA, USA). The SITO was separated on a 10 × 2.1-mm Waters XTerra RP18 Guard Column with a pore size of 5 µm. The chromatographic elution was accomplished with absolute methanol at a flow rate of 1 mL/min, and the injected sample volume was 50 μL. UV detection was set at 210.3 nm for SITO. The SITO loading capacity of liposomal β-sitosterol is expressed as: $\;\frac{\left[“concentration\;of\;SITO\;“\left(“after\;HPLC\;analysis”\right)”\;/\;total\;concentration\;of\;SITO”\right]}{\left[“concentration\;of\;EPC\;“\left(“after\;phosphorus\;assay”\right)”\;/\;total\;concentration\;of\;EPC”\right]}$ ×100%.

### 2.4. Cell Viability Assay

A number of cells (4 × 10^3^ CT26/*luc* cells per well) were seeded in a 96-well plate on the day before being treated with different concentrations of EPC, SITO, and LS for 72 h. The concentration of LS here indicated the concentration of the incorporated SITO. After the drug treatment, the medium was replaced with 100 μL of 3-[4,5-dimethylthiazol-2-yl]-2,5 diphenyltetrazolium bromide (MTT, 1 mg/mL, Sigma-Aldrich, St. Louis, MO, USA)- containing medium into each well and incubated at 37 °C for 4 h. Formazan was dissolved with dimethyl sulfoxide (DMSO, Sigma-Aldrich), and the absorbance was read using an ELISA reader (Bio-Tek Instruments, Winooski, VT, USA) with a wavelength of 570 nm. Cell viability $=\frac{the\;OD570\;of\;treated\;group}{the\;OD570\;of\;the\;control\;group}\times 100\%$.

### 2.5. Invasion Assay

Matrigel (BD Biosciences, Bergen, NJ, USA) was diluted with serum-free RPMI1640 medium at the ratio of 1:6. Transwell inserts with 8-μm pores (Corning Costar, Corning, NY, USA) were coated with 50 μL diluted Matrigel and incubated at 37 °C for an hour, allowing Matrigel to solidify. Some (5 × 10^4^ CT26*/luc*) cells were suspended in 200 μL of serum-free medium and then loaded on the upper chamber, and the upper chamber was transferred to the lower chamber filled with 700 μL of completed medium. The inserts were incubated at 37 °C for 20 h. Medium and noninvaded cells were removed with a cotton swab, and the filters were fixed with methanol/acetic acid (3:1) and then stained with hematoxylin. The invaded cells were counted under a light microscope (Leica Microsystems Incorporation, Wood Dale, IL, USA), and the control group was set as 100%.

### 2.6. Western Blotting

For the in vitro study, 2 × 10^5^ CT26/*luc* cells were cultured in a 10-cm dish, then treated with EPC (served as the control group), 16 μM SITO, or 16 μM LS for 72 h. For the in vivo study, the tumors were homogenized in the tissue protein extraction reagent (T-PER^TM^, Thermo Scientific, Waltham, MA, USA) containing 1% protease inhibitor cocktails (#P8340, Sigma-Aldrich). Supernatants were collected after the homogenates were centrifuged at 12,000× *g* at 4 °C for 15 min. Protein concentrations were assessed by the Bradford protein assay.

Seventy micrograms of total protein lysates were separated on 8% SDS-PAGE and transferred to a nitrocellulose membrane (P/N 66485, Life Sciences, Pall Corporation, Portsmouth, UK). The membrane was blocked with 5% nonfat milk in 1× TBST buffer solution (10 mM Tris-base, 150 mM NaCl, and 0.1% Tween 20) at room temperature for an hour, followed by incubation with the anti-MMP-9 (AB19016, Millipore, 1:1000, Burlington, MA, USA) and anti-β-actin (MABT523, Millipore, 1:1000) primary antibodies at 4 °C overnight. After washing and reacting the membranes with HRP-conjugated goat anti-rabbit antibody, the protein expression was detected using an enhanced chemiluminescence (ECL) system, and the signals were acquired using the Luminescence/Fluorescence Image System LAS-4000 (Fujifilm, Tokyo, Japan). Band intensities were quantified by ImageJ (National Institute of Health, Bethesda, MA, USA), and β-actin was used as the internal control for normalization.

### 2.7. Establishment of Lung Metastatic Mouse Model

The animal experiments complied with the institutional animal care and use guidelines (Protocol no. 1060811 was issued by the IACUC of the National Yang Ming Chiao Tung University, Taipei, Taiwan). Five to six-week-old male BALB/c mice were randomly assigned to three groups: PBS-treated control (Control), SITO (4 μmol/0.1 mL), and LS (4 μmol/0.1 mL) groups. A small amount (0.1 mL PBS or drugs) was given through oral gavage once a day for 7 days prior to CT26*/luc* cell injection. Some (2 × 10^5^ CT26*/luc*) cells were suspended in 0.1 mL serum-free RPMI 1640 medium and injected via tail veins to generate a lung metastatic mouse model. The mice continued to be treated with the same dosages twice a week throughout the entire experiment. The tumor metastases post-treatments were monitored from week 3 to week 5 by bioluminescent imaging (BLI). Mice were sacrificed at the end of the study, and the lungs and other organs and tissues were collected for ex vivo BLI, histopathological examination, and Western blot. The experimental details are shown in Supplemental Figure S2.

### 2.8. Assays of IL-12, IL-18, and IFN-γ in Small Intestines of Mice

Six-week-old male BALB/c mice were treated with PBS, SITO, or LS, as mentioned in the tumor model establishment section for 7 days prior to the sample collection. Mice were euthanized 3 h after the final administration, and the small intestines were removed and homogenized in the T-PER reagent containing 1% protease inhibitor cocktail. Homogenates were centrifuged at 12,000× *g* for 5 min, and the supernatants were collected and assayed with ELISA kits. The Mouse IL-12 p70 ELISA kit (M1270, R&D system, Minneapolis, MN, USA), mouse IL-18 ELISA kit (7625, MBL International, Nagoya, Japan), and mouse IFN-γ ELISA kit (MIF00, R&D system) were used to determine the IL-12, IL-18, and IFN-γ levels, respectively. The manufacturer’s instructions were followed, and the absorbance at 450 nm was measured using an ELISA plate reader (Bio-Tek).

### 2.9. Determination of T Cell Subsets by Flow Cytometry

The splenocytes of identical batches of mice used in the ELISA assay were isolated and stained with fluorescence-labeled antibodies to determine how the treatments (PBS, SITO, and LS) influenced the populations of CD4^+^ T cells and CD8^+^ T cells in mice. Briefly, 1 × 10^6^ splenocytes suspended in 1 mL of PBS were stained with FITC-conjugated anti-mouse CD3 (CD3-FITC, 100203, BioLegend, San Diego, CA, USA), phycoerythrin-conjugated anti-mouse CD4 (CD4-PE, 100407), peridinin chlorophyll protein complex-conjugated anti-mouse CD8, and (CD8-PerCP, 100731, BioLegend) monoclonal antibodies. The percentages of the CD4^+^ and CD8^+^ T-cell subsets were determined using a FACSCalibur flow cytometer (BD Bioscience).

### 2.10. In Vivo and Ex Vivo Bioluminescent Imaging (BLI)

In vivo and ex vivo BLI were performed with an IVIS50 Imaging System (Xenogen, Alameda, CA, USA). Fifteen minutes prior to in vivo imaging, tumor-bearing mice were intraperitoneally injected with *D*-luciferin (150 mg/kg in PBS) and anesthetized using 1–3% isoflurane. For ex vivo imaging, organs and tissues harvested from tumor-bearing mice were incubated in *D*-luciferin (3 mg/mL)-containing PBS. Regions of interest (ROIs) were drawn around the tumors, organs, or tissues, and the acquired signals were quantified as photons/sec with Living Image software (version 2.20, Xenogen).

### 2.11. Histopathological Examination

The standard protocol was followed to conduct hematoxylin and eosin (H&E) staining. Tumors, organs, and tissues harvested from mice were immediately fixed in 4% paraformaldehyde and embedded in paraffin. The tissue blocks were sectioned with 5-μm thickness. After deparaffinization and rehydration, the slides were stained for general morphologic evaluation. Briefly, tissue sections were stained with hematoxylin solution for 2 min, followed by five washes with tap water, 5 dips in 1% acid ethanol (1% HCl in 70% ethanol), and rinsed with distilled water. The sections were then stained with eosin solution for 30 s, followed by dehydration with graded alcohol and cleaned with xylene. The mounted slides were examined and photographed using an Olympus BX61 fluorescence microscope (Tokyo, Japan).

### 2.12. Statistical Analysis

The Student’s *t*-test was used to determine whether there was a significant difference between the two groups. The differences among multiple groups were analyzed using one-way ANOVA and Tukey’s method, as chosen for the post hoc test. All data were presented as the mean ± standard error (SE). The differences were considered significant when *p* < 0.05.

## 3. Results

### 3.1. Characterization of Liposomal β-Sitosterol

**Figure 1:**
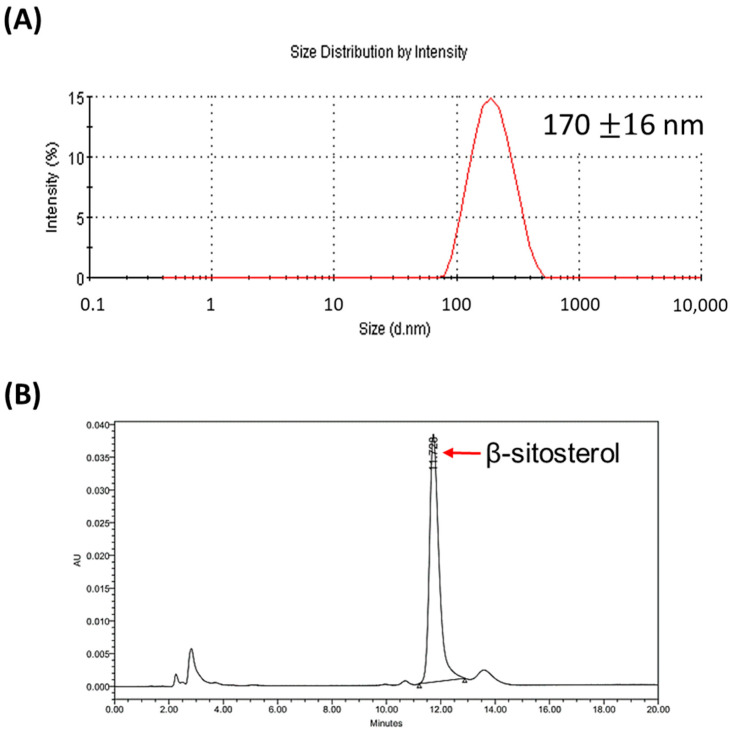
Characteristics of liposomal β-sitosterol (LS). (**A**) DLS analysis determined the average size of LS at 170 ± 16 nm. (**B**) The UV absorption spectrum of β-sitosterol obtained from HPLC analysis was used to calculate the concentration of β-sitosterol in LS.

Liposomalβ-sitosterol (LS) was prepared using the thin-film method. Malvern Zetasizer Nano was used to determine the size of the LS. The mean hydrodynamic size of the extruded LS was in the range of 170 ± 16 nm (Figure 1A). The concentrations of the incorporated EPC and SITO in the LS were determined by the Rouser’s method and HPLC. Figure 1B shows a representative chromatogram of the SITO detected by HPLC. The concentrations of EPC and SITO were 46.8 and 22.1 μmol/mL, respectively, and the loading capacity was 94.4%, calculated as the incorporated amount of SITO in liposomes.

### 3.2. β-Sitosterol Suppresses Cell Growth and Invasion of CT26/luc Cells

**Figure 2:**
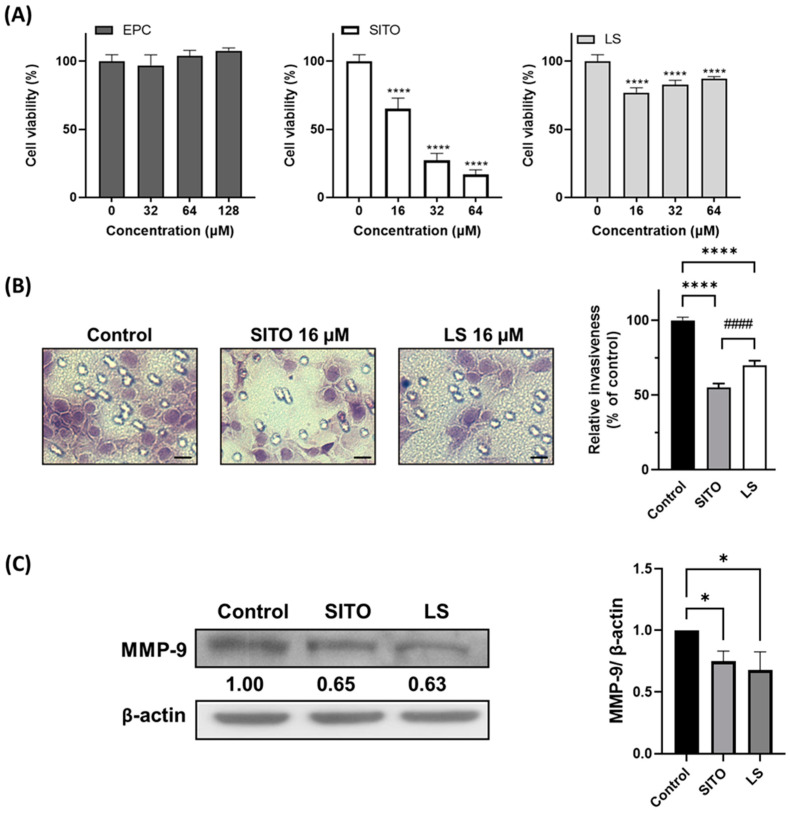
Changes in cell viability, invasiveness, and MMP-9 of the CT26/*luc* cells caused by different treatments. (**A**) CT26/*luc* cells were treated with various concentrations of EPC, β-sitosterol (SITO), and liposomal β-sitosterol (LS) for 72 h and subjected to the MTT assay. Each point represents the mean ± SE of three independent experiments. (**B**) Invasiveness of the CT26/*luc* cells was determined using Matrigel-coated Transwell inserts after being treated with the vehicle, SITO, and LS for 3 days. The vehicle-treated controls were set as 100%. Photos were taken with 40× magnification. Scale bar = 20 µm. (**C**) Expression of MMP-9 in the vehicle, SITO, and LS-treated CT26/*luc* cells was analyzed by Western blot. All experiments were independently performed three times. (* *p* < 0.05 and **** *p* < 0.0001 compared with the Control; #### *p* < 0.0001 compared with SITO).

The cytotoxicity of the EPC, SITO, and LS to CT26/*luc* cells were evaluated by the MTT assay 72 h after treatment. EPC did not cause cytotoxicity to the CT26/*luc* cells, indicating that EPC should be a safe material for making liposomes. Significant cytotoxicity was observed for cells treated with 16 μM of free SITO. In contrast, 64 μM of LS did not result in substantial cell death in CT26/*luc* cells compared with the cytotoxicity caused by the SITO treatment (Figure 2A).

Distal metastasis is often observed in patients with colorectal carcinoma. Therefore, an invasion assay was performed to evaluate the migration inhibition of SITO and LS in CT26/*luc* cells. Figure 2B shows that invasion was significantly reduced in cells treated either with SITO or LS compared to that of the Control group (*p* < 0.001). The Control group was normalized as 1, and the percentages of invaded cells were 55% and 70% for SITO and LS, compared to 100% of the Control group. Antimigration caused by the SITO treatment was more significant than that of the LS group (*p* < 0.001). Since MMP-9 plays an important role in tumor cell invasion, we further assayed the expression of this protein with Western blotting (Figure 2C). Both SITO and LS decreased the MMP-9 expression 0.65- and 0.63-fold compared with the Control group.

### 3.3. Liposomal β-Sitosterol Suppresses Tumor Progression In Vivo

**Figure 3:**
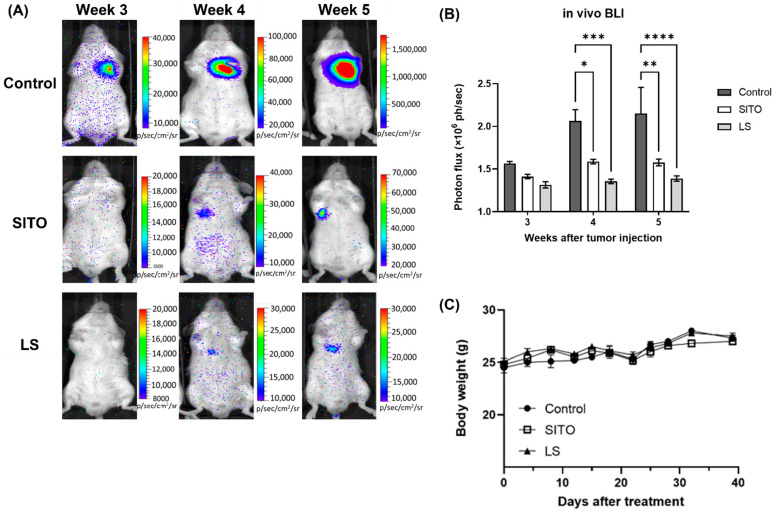
BLI showed the tumor progression in vivo. (**A**) BLI monitored the progression of lung metastasis noninvasively in mice receiving different treatments in vivo. (**B**) The quantitative results of BLI demonstrated that the SITO and liposomal β-sitosterol (LS) groups showed better tumor suppressions compared with that of the Control group from week 3 to week 5 after cell injection. (**C**) The body weight monitoring post-treatments up to 40 days. The body weight changes were within 20% among the three groups, suggesting no general toxicity post-SITO or LS treatments. Each point represents the means ± SE. (* p < 0.05, ** *p* < 0.01, *** *p* < 0.001, and **** *p* < 0.0001).

CT26/*luc* cells were injected into BALB/c mice via the tail veins to generate a lung metastasis model and received different treatments, and the tumor progression was monitored by bioluminescent imaging (BLI). Representative BLI images obtained from week 3 to week 5 after CT26/*luc* cell injection are shown in Figure 3A, demonstrating that the LS group had the lowest BLI signals in the lungs among all groups. BLI quantification of all three groups are shown in Figure 3B. Notably, the average BLI signals of the Control group increased with time after the tumor injection but were not observed in the SITO and LS groups. Both the SITO and LS groups had significantly lower BLI signals than the Control group from week 4. The changes in body weight were within 20% for all groups over the whole experimental period, suggesting no or minimal toxicity caused by the SITO and LS treatments, as shown in Figure 3C.

**Figure 4:**
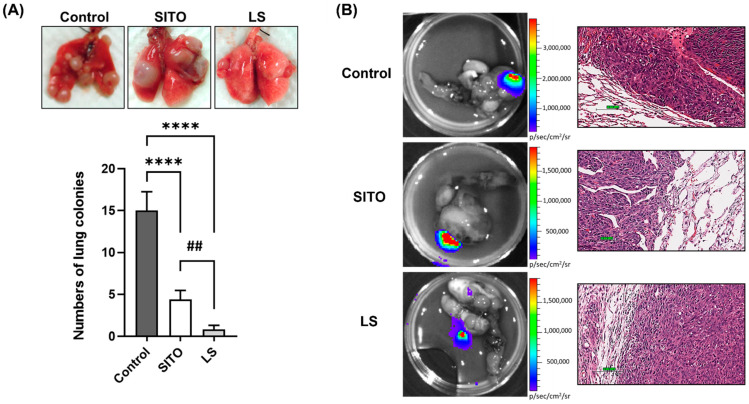
Liposomal β-sitosterol (LS) strongly repressed tumor progression in the lungs. (**A**) Representative images of lungs from the Control, SITO, and LS-treated mice. Lungs were removed after the last BLI, and the number of metastatic lung colonies was counted. Mice treated with LS had the fewest lung colonies compared with the Control and SITO groups. (**B**) Representative images of H&E-stained lung sections from animals (40× magnification). Scale bar = 100 µm. (**** *p* < 0.0001 compared with the Control; ## *p* < 0.01 compared with SITO).

The mice were euthanized on week 6 after BLI. Various tissues and organs, including lungs, were removed and examined by ex vivo BLI. Digital photos of the excised lungs were taken, and the number of lung colonies was counted (Figure 4A). Mice treated with LS had significantly fewer lung colonies than those of the Control and LS groups, and the SITO group also had fewer lung colonies than that of the Control group. Both BLI and histopathological images of the excised lungs are shown in Figure 4B. The histopathological findings further confirmed the BLI results.

**Figure 5:**
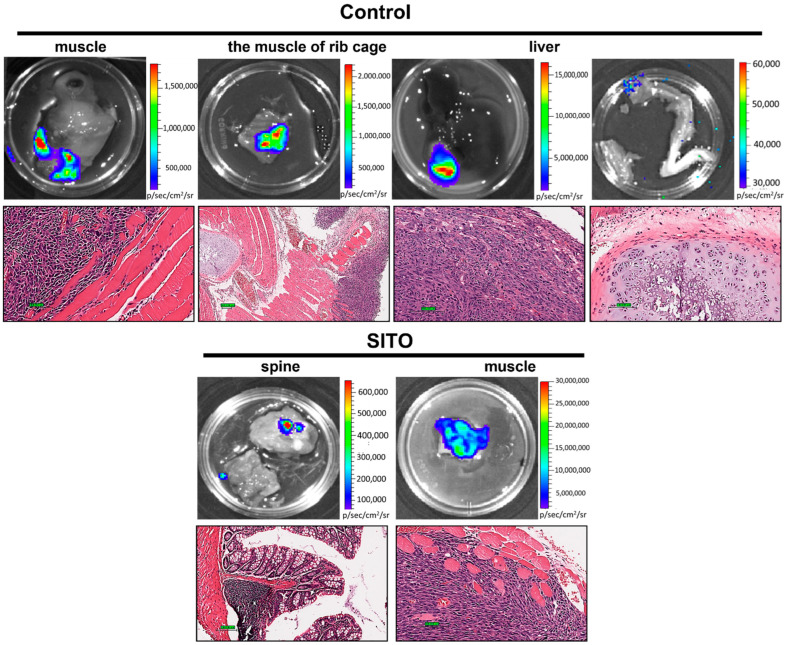
Liposomal β-sitosterol (LS) treatment effectively suppresses distal metastases in mice receiving an intravenous injection of CT26/*luc* cells, as presented by ex vivo BLI and a histopathologic analysis. Ex vivo BLI was performed on 11 different tissues excised from each mouse after the final BLI, and the metastases were validated by H&E staining. Scale bar = 100 µm.

Ex vivo BLI was performed on 11 different tissues and organs excised from each mouse after the final in vivo BLI on week 6 (Figure 5). Only the tissues or organs derived from the Control and SITO groups demonstrated positive BLI signals and were further examined with histopathology. Most mice in the Control group exhibited bioluminescent evidence of metastases in the lungs, muscle, muscle of the rib cage, liver, and bone. SITO-treated mice also showed some metastases in the spine and muscle. In contrast, all LS-treated mice had no evidence of metastasis other than the lungs. The fraction of mice with metastatic lesions in each group is presented in Table 1.

### 3.4. Liposomal β-Sitosterol Treatment Elevates the Expressions of IL-12, IL-18, and IFN-γ in the Small Intestine and Increases the CD4^+^/CD8^+^ T Cell Subsets

**Figure 6:**
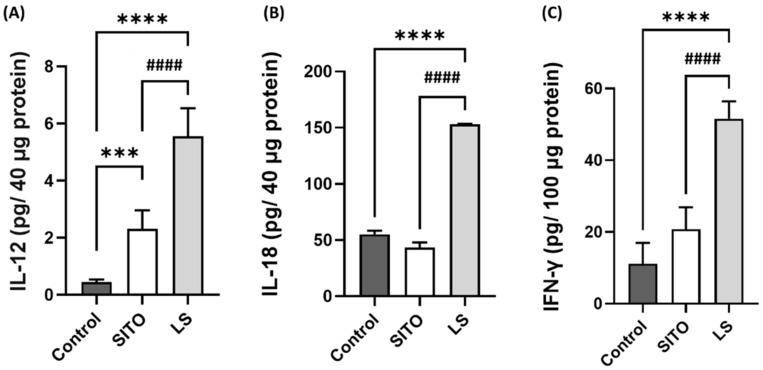
Liposomal β-sitosterol (LS) elevates the expression of IL-12, IL-18, and IFN-γ and inhibits the expression of MMP-9 in vivo. Mice were orally administered with PBS, β-sitosterol (SITO), or LS. The small intestines of mice were removed 3 h post the last treatment, and the proteins were extracted and subjected to the ELISA assay. The levels of (**A**) IL-12, (**B**) IL-18, and (**C**) IFN-γ were determined using an ELISA kit according to the manufacturer’s protocols. The data were shown as the means ± SE. (*** *p* < 0.001 and **** *p* < 0.0001 compared with the Control; #### *p* < 0.0001 compared with SITO).

Another in vivo experiment was performed to investigate the correlation between the immune response and tumor suppression. Mice were treated with PBS, SITO, or LS by oral gavage for a week before sample collection. The small intestines were removed from mice 3 h post-the last dose of treatments, and the proteins were extracted for the assay of the IL-12, IL-18, and IFN-γ levels. Compared with those of the Control and SITO groups, the IL-12, IL-18, and IFN-γ levels in the LS group were all significantly elevated (Figure 6A–C). A significant increase in the IL-12 level was also detected in the SITO group compared with the Control group.

Moreover, the spleens of mice were also removed for collecting splenocytes. The splenocytes were stained with specific antibodies to evaluate the percentage of CD4^+^/CD8^+^ T-cell populations post-treatments. As shown in Table 2, significant increases in CD4^+^ and CD8^+^ T-cell subsets were found in the LS group compared with those of the Control and LS groups. The SITO treatment also led to significant increases in CD4^+^ and CD8^+^ T-cell subsets compared to the Control group.

## 4. Discussion

In addition to lowering the serum cholesterol level [23], phytosterol-rich diets also reduce the risk of cancers in the esophagus, ovary, and colorectum [24,25,26]. As a member of the phytosterol family, β-sitosterol (SITO) has been reported to exert the chemopreventive and chemoprotective capabilities against cancer progression and metastasis [27,28]. However, it has been suggested that the bioavailability of phytosterols is only 0.5–2%, indicating that huge amounts of phytosterols are required to achieve an effective concentration [29]. Using the liposome as the carrier to deliver phytochemicals has been proposed and shown to improve the uptake in the designated tissues [30]. For these reasons, the main purpose of this study was to investigate the anticancer effects of liposomal β-sitosterol (LS) on tumor progression and metastasis using CT26/*luc* tumor-bearing mice.

Liposomes with an average size of around 200 nm increase the uptake in the intestines efficiently [31]. Liposomes have also been shown to target M cells and Peyer’s patch after oral administration [32]. The drug loading capacity is another critical factor that needs to be considered when using liposomes as drug carriers for anticancer therapy. The influence of several liposome formulation parameters on the encapsulation, stability, loading capacity, and drug release with glibenclamide liposomes has been proposed [33]. In this study, liposomal β-sitosterol was successfully prepared. The size and the loading capacity of liposome was 179 nm (Figure 1A) and 94.4%, respectively. Poor solubility and bioavailability of free phytosterols sometimes leads to controversial results between in vitro and in vivo studies, suggesting the importance of liposomal encapsulation. Some studies have shown that liposomal drugs have a better water solubility and enhanced bioavailability than free drugs do in vivo. As shown in this study, LS resulted in a lower cytotoxicity than free SITO (Figure 2A) when the same concentrations of SITO were applied to CT26/*luc* cells. SITO showed a dose-dependent cytotoxicity with the lower cell viability at higher doses, while the cytotoxicity of LS did not increase as the doses of LS increased. These results imply that liposomal encapsulation may reduce the release of SITO when the liposomes come into contact with the medium and cells [34] and may decrease the nonspecific toxicity to any tissues. SITO-based liposomes displayed a higher drug retention than that of cholesterol-based liposomes [35,36]. SITO-containing liposomes (20 mol%–33 mol%) released 15–30% of the drug after incubating at a neutral pH buffer for 48 h to 14 days. Therefore, the concentration of free SITO released from LS might be much lower than expected and result in no significant difference in cytotoxicity among different LS treatments. Accordingly, the liposome can be a good candidate as the drug carrier of SITO.

β-Sitosterol has been reported to suppress tumor proliferation and dissemination in multiple cancer types [9], including colorectal cancer [36,37]. Liposomal β-sitosterol also has been shown with a chemopreventive effect on tumor metastasis [38]. Our results showed that both SITO and LS were equally effective in repressing the invasiveness of CT26/*luc* cells, possibly through inhibiting MMP-9 expression (Figure 2B,C). These results are similar to those observed in breast and pancreatic cancers [39,40]. MMP-9 expression is strongly correlated to cell migration and invasion. MMP-9 is regulated by NF-κB, and SITO has been shown to suppress NF-κB and its downstream proteins in LPS-stimulated BV2 cells [6]. Here, a psuedometastatic CT26/*luc* tumor-bearing animal model established by intravenous injection of CT26/*luc* cells was chosen to validate whether SITO and LS have antimetastatic ability. CT26 cells were derived from immunocompetent Balb/c mice and showed high immunogenicity; therefore, the CT26 syngeneic tumor model is widely used to evaluate the efficacy of immunotherapy [41]. Moreover, the pseudo-metastatic CT26 animal model is also utilized in antimetastatic studies [42]. Both BLI and the ex vivo BLI results show that the LS group had more inhibition on lung metastases than the Control and SITO groups (Figure 3).

Except for the lung colonization (Figure 3 and Figure 4), metastases to other distal organs were not found in the LS group (Figure 5). Notably, we observed muscle metastases in some mice. Muscle metastases are rarely seen in cancer patients, with inconsistent probability ranges from 0.03 to 17.5% [43]. Muscle metastases may result from adjacent subcutaneous tissues and bone or through hematogenous spreading [44]. Here, we found that the CT26/*luc* cells metastasized to those muscles were mainly found in the dorsal area close to the rib region.

β-Sitosterol has been reported to modulate immune responses in several studies. SITO demonstrated a protective immune response via the induction of Th1 against disseminated candidiasis by increasing the IL-2 and IFN-γ expressions in mice [45]. SITO treatment could increase IL-6, TNF-α, MCP-1, and IFN-γ production in lipopolysaccharide (LPS)-treated macrophages [46]. Liposomal β-sitosterol has been shown to inhibit metastasis by enhancing IL-12 and IL-18 expressions in vivo [40]. Here, we also found that the IL-12, IL-18, and IFN-γ levels were significantly increased in the small intestines of LS-treated mice compared with those of the Control and SITO groups (Figure 6). Table 2 demonstrates that the CD4^+^/CD8^+^ T-cell populations are increased in the LS group compared to the other two groups. The proliferation of peripheral blood mononuclear cells and activation of dendritic cells are also found elevated in pigs treated with SITO [47]. Though IL-18 is not directly related to the development of Th1 cells, it cooperates with IL-12 to activate Th1 cells to generate IFN-γ. Hence, IL-18 is a cytokine that enhances antitumoral type 1 responses [48]. IL-12 and IL-18 could skew T cells towards antitumoral Th1 types synergistically when present simultaneously [49]. In addition, the number of tumor-infiltrating T cells strongly correlates with favorable outcomes in breast and gastric cancers [50,51].

Cancer metastasis accounts for more than 90% of cancer deaths and presents an ongoing challenge for modern drug discovery. An intravenously injected method has been employed in many cancer-bearing animal models for preclinical metastatic studies [52]. Advances in optical imaging technology provide excellent opportunities for researchers to develop animal disease models that recapitulate the complexities of human cancer progression [53]. BLI was applied in the CT26/*luc* model to track metastasis in living mice in the current study. The BLI results showed significant tumor inhibition in the LS-treated group, suggesting that BLI is a powerful tool for studying these disseminated diseases. Lastly, the gut microbiota has become an active research field recently. β-sitosterol has been shown to exert the potential to modify the gut microbiota and even inhibit colorectal cancer progression [54,55,56]. The liposomal β-sitosterol generated and utilized in the current study are mainly taken up by the intestinal epithelium and may change the gut microbiota, which is worth further investigation. Based on the results reported by Imanaka et al., SITO-based liposomes (LS) showed a lower accumulation in the liver and intestinal epithelium after oral administration compared to the cholesterol-based liposomes. Massive liver accumulation and possible liver toxicity are the bottlenecks when using liposomes for drug delivery. Based on these findings, orally administered LS might lower the liver uptake and increase the uptake levels of hydrophobic molecules in the targeted tissues. Therefore, the accumulation mechanisms and the pharmacokinetics should be elucidated using strategies like conjugating radioisotopes to LS in the future.

In conclusion, liposomal β-sitosterol significantly reduces the lung and distal tissue/organ metastases in CT26/*luc* tumor-bearing mice compared with the Control and β-sitosterol groups. Both the in vitro and in vivo results validate that the mechanisms behind the LS-mediated anticancer effects are related to MMP-9 inhibition and the activation of antitumoral immunity.

## Figures and Tables

**Table 1:** The number of metastatic lesions was determined using a combination of ex vivo BLI and a histological analysis of all mice upon sacrifice.

Metastasis Site	Control	SITO	LS
Lungs	9/10	5/10	3/10
Muscle	4/10	2/10	0/10
Bone	1/10	1/10	0/10
Liver	1/10	0/10	0/10
Total metastatic lesions	15	8	3

**Table 2:** Percentages of CD4^+^ and CD8^+^ T-lymphocyte subsets in the spleens of the control, β-sitosterol (SITO)-, and liposomal β-sitosterol (LS)-treated mice. Percentage of CD4^+^/CD8^+^ T-lymphocyte subsets expressed as the means ± SE. * *p* < 0.05 and ** *p* < 0.01 as compared to that of the control. Significant differences between SITO and LS were expressed as # *p* < 0.05.

Group	CD4^+^ T Cells (%)	CD8^+^ T Cells (%)
Control	29.0 ± 0.8%	45.8 ± 1.8%
SITO	31.7 ± 0.4% *	50.5 ± 0.8% *
LS	34.7 ± 0.6% **,^#^	53.7 ± 0.7% **,^#^

## Data Availability

Data are contained within the article or supplementary materials.

## Supplementary Materials

The following supporting information can be downloaded at https://www.mdpi.com/article/10.3390/pharmaceutics14061214/s1: Figure S1: The correlation between the emitted photon flux and cell numbers of the CT26/*luc* cells. Figure S2: The details of the in vivo experiment.
