# Supplementary material for: Liposomal β-Sitosterol Suppresses Metastasis of CT26/luc Colon Carcinoma via Inhibition of MMP-9 and Evoke of Immune System
Source: Pharmaceutics. 2022 Jun 7;14(6):1214. doi: 10.3390/pharmaceutics14061214 (PMC9231002; doi:10.3390/pharmaceutics14061214)
Supplement: Supplementary file 1 [file pharmaceutics-14-01214-s001.zip › pharmaceutics-1711394-supplementary.pdf]

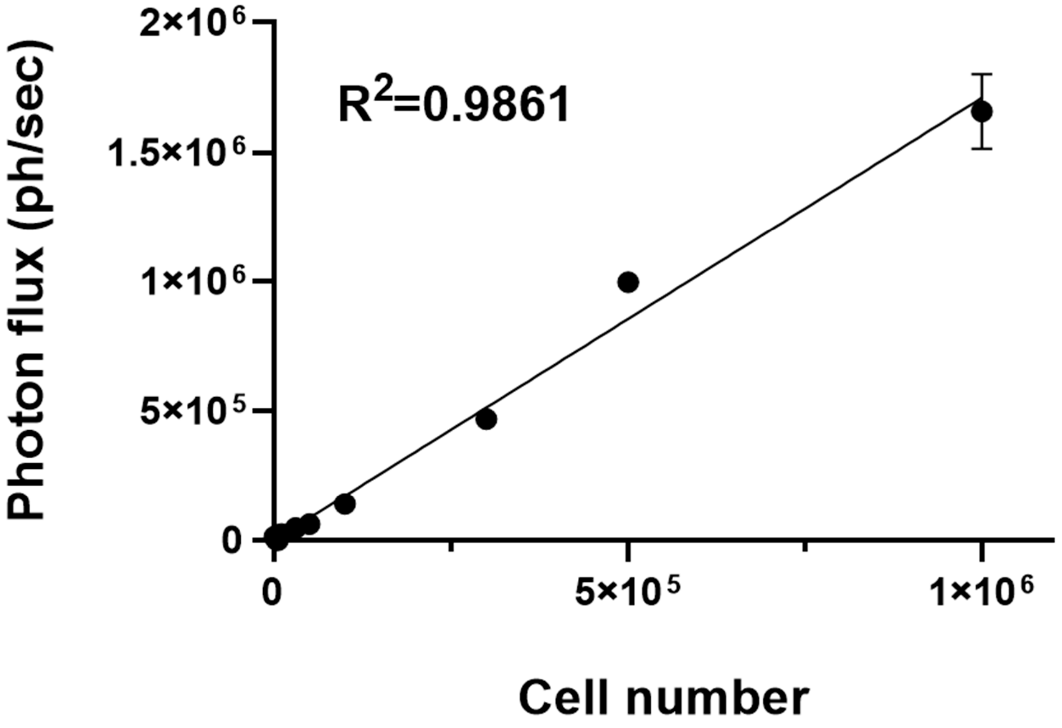

Supplementary Figure 1. The correlation between cell numbers and photon flux of CT26/luc cells. Different numbers of CT26/luc cells were plated in a 96-well plate and treated with *D*-luciferin. Emitted photons were acquired by IVIS50 and quantified as photon flux (photons/sec). The correlation curve of cell numbers and emitted photon flux was established with R^2=0.9861.

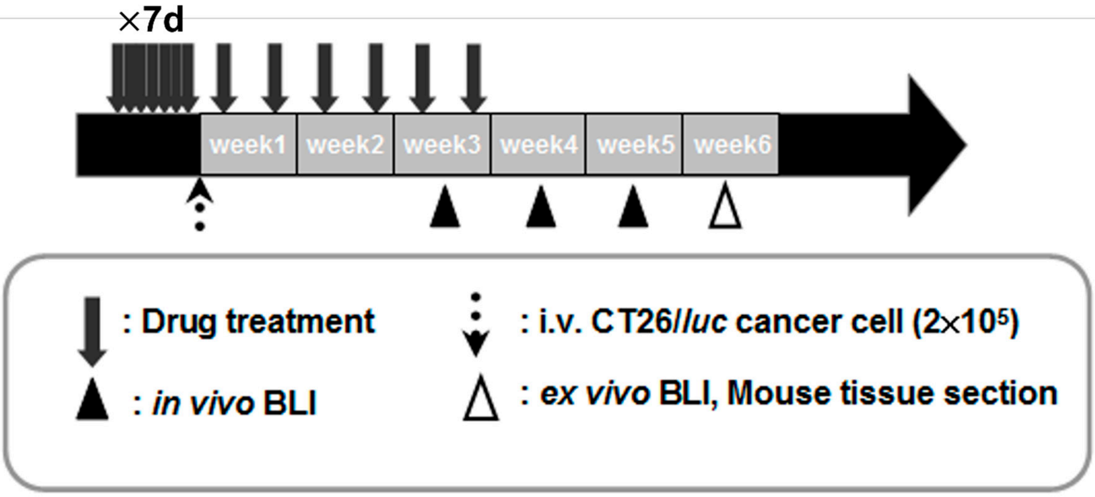

Supplementary Figure 2. The details of *in vivo* experiment. Male BALB/C mice received 7-day treatments prior to the intravenous injection of CT26/*luc* cells. Different treatments were continued throughout the study, and BLI was utilized to monitor tumor progression from week 3 to week 5. The mice were euthanized on week 6, and organs/tissues were collected for ex vivo BLI and histopathological analysis.
